# Our children are at risk of COVID-19- associated rhino-orbito-cerebral mucormycosis (ROCM)

**DOI:** 10.1016/j.amsu.2021.103058

**Published:** 2021-11-16

**Authors:** AbdulRahman A. Saied, Asmaa A. Metwally, Kuldeep Dhama

**Affiliations:** a Department of Food Establishments Licensing (Aswan Branch), National Food Safety Authority (NFSA), Aswan, 81511, Egypt; b Touristic Activities and Interior Offices Sector (Aswan Office), Ministry of Tourism and Antiquities, Aswan, 81511, Egypt; c Department of Surgery, Anesthesiology, and Radiology, Faculty of Veterinary Medicine, Aswan University, Aswan, 81511, Egypt; d Division of Pathology, ICAR-Indian Veterinary Research Institute, Izatnagar, Bareilly, Uttar Pradesh, India

**Keywords:** COVID-19, SARS-CoV-2, Children, Rhino-orbito-cerebral mucormycosis, Surgery, Risk

COVID-19 associated mucormycosis (CAM) has been widely reported in adults and came into the limelight particularly during the second wave of COVID-19 in India [1,2]. Predisposing risk factors responsible for rapid surge in CAM cases in India have been reported to be diabetes mellitus, overdoses of steroids, high iron levels and immunosuppression along with lack of hygienic conditions, longer hospitalization, use of ventilators and leaky humidifiers in oxygen cylinders [3]. Mucormycosis a life-threatening fungal infection that can present as a local or systemic invasion [4]. Many saprophytic, ubiquitous fungi of the order *Mucorales*, subphylum *Mucoromycotina*, and class *Zygomycetes*, such as *Rhizomucor*, *Rhizopus*, *Apophysomyces*, *Mucor*, *Saksenaea*, or *Lichtheimia*, cause mucormycosis [5]. *Rhizopus oryzae* is the most common etiologic agent of ROCM, and it's also the most common manifestation that starts in the nasal turbinates [6]. CAM has recently been identified in various countries, notably among individuals with uncontrolled diabetes and steroid usage [1,7,8]. It is a deadly fungal disease reported in adults with a high mortality rate and devastating impacts, hence optimal glycemic control, limiting injudicious use of steroids, early confirmatory diagnosis, and multidisciplinary team management with systemic antifungals, surgical debridement, controlling of comorbidities are crucial in saving lives of such patients [[8], [9], [10], [11]]. Currently, mucormycosis has also been reported to be increasing in diabetic pediatric patients associated with SARS-CoV-2 infection, with fatal outcomes [12,13].

Apart from adults, children are also being infected with SARS-CoV-2 and its emerging variants during the COVID-19 pandemic [14]. Clinical symptoms of SARS-CoV-2 are linked to age [15]. Adults experience respiratory symptoms, the most severe cases can lead to acute respiratory distress syndrome (ARDS) and multiple organ manifestations, whereas children seldom show significant respiratory symptoms and often remain asymptomatic, however, SARS-CoV-2 infection in children can lead to severe disease and can develop a life-threatening multisystem inflammatory syndrome (MIS-C) [[14], [15], [16], [17], [18]]. There are current endeavors for developing an effective vaccine and testing the current available vaccines for their suitability in kids to protect them against SARS-CoV-2 and its newly emerged variants of concern, such as delta variant (called Indian variant) and others are surging, characterized by higher transmissibility, virulence and causing vaccine break-through events [[19], [20], [21]]. The extraordinary worldwide surge in instances of COVID-19 related mucormycosis drove medical health to the edge even before the assault of the ongoing COVID-19 pandemic could settle.

Alongside the SARS-CoV-2 lethal impact and the diabetogenic condition [22,23] of COVID-19, our children with diabetes type 1 are at risk of being infected with mucormycosis, the black fungus disease. Diabetic ketoacidosis (DKA) is considered an excellent medium that facilitates *Mucorales* spores to germinate in patients with COVID-19. This fungus thrives in an environment of elevated glucose and acid pH, as it has an active ketone-reductase system [24]. The high fungal spore load in the Indian environment, besides the immunosuppression induced by COVID-19 itself, systemic steroids and anti-Interleukin-6 agents (e.g, tocilizumab), provides a suitable environment for invasive fungal diseases in our children as susceptible hosts [12].

Rhino-orbital cerebral mucormycosis (ROCM) is a rare but rapidly progressing disease that can result in death if not recognized and treated timely and aggressively, and this fungal disease spread like an epidemic within the COVID-19 pandemic [25]. Uncontrolled diabetes is just a predisposing factor in 15% of pediatric patients. Early diagnosis based on clinical symptoms and biopsy is highly suggested to avoid and minimize fatality rates from this serious disease [13]. In India, an alarming rise in ROCM in post-COVID cases has been documented in adult patients with uncontrolled diabetes. Diwakar J et al., [12] have also reported the first cases of COVID-19-associated ROCM in two pediatric patients with Type 1 diabetes mellitus. The fungal culture revealed the infection with *Rhizopus arrhizus*. Both patients had an asymptomatic SARS-CoV-2 infection and developed ROCM while receiving therapy for diabetic ketoacidosis. They haven't received steroids therapy. Cavernous sinus thrombosis was seen in both instances, with involvement of the orbit, paranasal sinuses, and brain. The patients had undergone a craniotomy with abscess drainage. The patients were given liposomal amphotericin B (LAMB) and systemic antibiotics after the surgery. Both of them finally had to go through orbital exenteration, the surgical removal of the eyeball and the surrounding tissues.

Due to a combination of viral and drug-induced immunosuppression, these pediatric individuals are at an elevated risk of secondary infections, such as ROCM, one of the fiercest diseases in terms of symptoms and treatment. ROCM is a fast progressing illness, and early diagnosis combined with vigorous surgery and antifungal treatment can save lives. Additionally, the neurological manifestations of ROCM in the pre-and post-COVID-19 era are almost similar. However, there have been few instances of ROCM neurological symptoms in the past [26].

It is critical to eliminate underlying risk factors when treating mucormycosis (glycemic management, glucocorticosteroid tapering, reduction or discontinuation of immunosuppressive drugs). Recently, diabetes, steroids and remdesivir were not found to be associated with increased mortality risk in CAM patients, therefore the use of steroids to manage critical COVID-19 patients can be continued. The involvement of lungs, bilateral manifestation, and isolation of *Rhizopus* isolation are responsible for increased mortality risk, necessitating proactive screening in severely ill patients [27]. The medical treatment of choice is a systemic antifungal medication with liposomal Amphotericin B; however, drug resistance is not uncommon. Devitalized tissue frequently requires surgical debridement. Children with mucormycosis should receive high doses of LAMB and surgical intervention as soon as possible [[28], [29], [30]]. Furthermore, despite its nephrotoxicity, scarcity, and expensive price, L-AMB is an antifungal medicine that has been utilized for the prevention, prophylactic and treatment of fungal infection in immunocompromised children [31]. Surgical management and drugs like amphotericin B and posaconazole have been found effective in reducing mortality risks [27]. Physicians should have a high index of suspicion, especially in post-COVID or symptomatic patients with uncontrolled diabetes, to avoid a delayed diagnosis. Any diabetic pediatric patient who exhibits respiratory symptoms such as coughs or develops a skin lesion should be thoroughly checked for mucormycosis, and treatment should be started as soon as possible. Using antigen rapid diagnostic tests (Ag-RDTs) alongside prevention strategies can be easily optimized for schools and college campuses [32]. We should take into our consideration the balance between the safety and quality of our children life when using the prevention strategies. SARS-CoV-2 infection in children has different clinical symptoms than in adults. Immune response in children is developed regardless of developing MIS-C. Children can have gastrointestinal symptoms rather than respiratory symptoms, and the virus can be found in their faces for a long time [33]. Therefore, developing age-targeted strategies for testing and protecting the population is necessitated amid the ongoing COVID-19 pandemic and subsequent threats of mucormycosis in affected patients [34].

## Author contributions

All the authors contributed significantly in this manuscript. AAS conceptualized the manuscript. AAS wrote the first draft with input from AAM, and KD reviewed and updated the manuscript. All authors contributed to revisions and approved the final manuscript.

## Provenance and peer review

Not commissioned, externally peer-reviewed.

## Sources of funding

None.

## Ethical approval

Not applicable.

## Guarantor

AbdulRahman A Saied.

## Declaration of competing interest

The authors declare that there is no conflict of interests.
